# Congenital myasthenic syndromes: increasingly complex

**DOI:** 10.1097/WCO.0000000000001300

**Published:** 2024-07-25

**Authors:** Sithara Ramdas, David Beeson, Yin Yao Dong

**Affiliations:** a MDUK Neuromuscular Centre, Department of Paediatrics, University of Oxford; b Department of Paediatric Neurology, John Radcliffe Hospital; c Neurosciences Group, Weatherall Institute of Molecular Medicine, The John Radcliffe, Oxford OX3 9DS

**Keywords:** acetylcholine receptor, congenital myasthenic syndrome, neuromuscular junction, soluble N-ethylmaleimide-sensitive factor attachment, treatment

## Abstract

**Purpose of review:**

Congenital myasthenia syndromes (CMS) are treatable, inherited disorders affecting neuromuscular transmission. We highlight that the involvement of an increasing number of proteins is making the understanding of the disease mechanisms and potential treatments progressively more complex.

**Recent findings:**

Although early studies identified mutations of proteins directly involved in synaptic transmission at the neuromuscular junction, recently, next-generation sequencing has facilitated the identification of many novel mutations in genes that encode proteins that have a far wider expression profile, some even ubiquitously expressed, but whose defective function leads to impaired neuromuscular transmission. Unsurprisingly, mutations in these genes often causes a wider phenotypic disease spectrum where defective neuromuscular transmission forms only one component. This has implications for the management of CMS patients.

**Summary:**

Given the widening nonneuromuscular junction phenotypes in the newly identified forms of CMS, new therapies need to include disease-modifying approaches that address not only neuromuscular weakness but also the multisystem involvement. Whilst the current treatments for CMS are highly effective for many subtypes there remains, in a proportion of CMS patients, an unmet need for more efficacious therapies.

## INTRODUCTION

**FIGURE 1:**
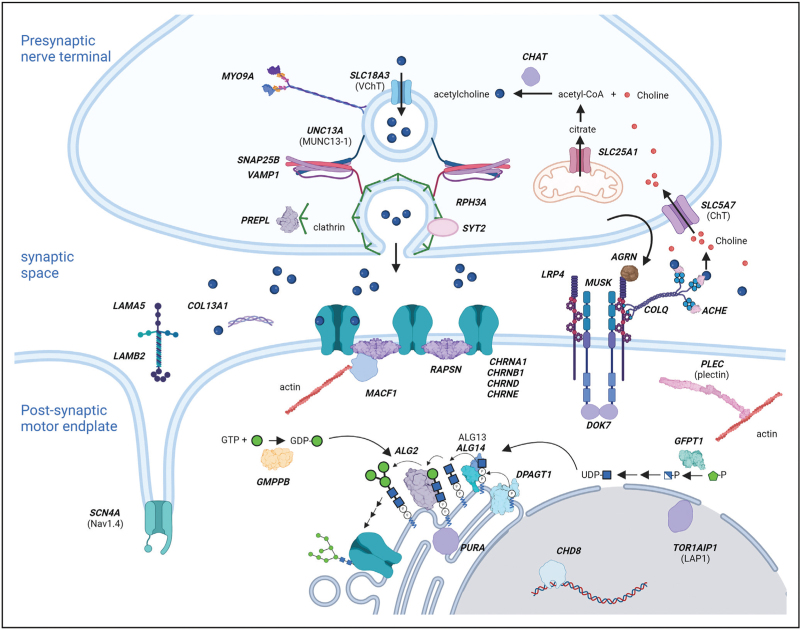
Schematic diagram of how the different congenital myasthenia syndromes-associated gene products are involved in neuromuscular junction biology, based on our current understanding. Gene names are in bold italicised capitals. Where the protein names are different to the gene names, they are in brackets beneath the gene name.

Congenital myasthenia syndromes (CMS) are caused by genetic defects that impair neuromuscular transmission. Efficient synaptic transmission at the neuromuscular junction (NMJ) incorporates many different proteins involved both in the signal transmission itself and in generating the prerequisite specialized synaptic structures. As a consequence, there are multiple pathogenic mechanisms that can underlie CMS. A recent comprehensive review of CMS highlights many of these mechanisms [1^▪▪^]. There are currently 35 genes reported to be associated with CMS (Fig. 1 and Table 1). The prevalence reported varies considerably between regions, 2.8–14.8 per million (UK), 1.8 per million (Brazil), and 22.2 per million (Slovenia) [2]. Differences are likely to occur because of mutation founder effects in certain regions and historically because of different pick-up rates as new detection technologies are adopted. The clinical use of next-generation sequencing greatly facilitates detection of mutations in larger genes, such as *AGRN*, *LRP4*, *MUSK*, *COLQ*, and thus increases in reported prevalence is likely to continue [3–7]. Whilst symptom onset at birth or infancy is the commonest presentation, later onset in childhood and adult life is now increasingly recognized. The clinical phenotypes can vary in the subtypes, but the hallmark is fatigable weakness predominantly affecting axial and proximal limb muscle, ocular, bulbar and or respiratory involvement. A suspected diagnosis is based on typical clinical features, decremental responses of 10% or more on repetitive nerve stimulation (RNS) and/or abnormal jitter and block on single-fibre EMG. It is prudent to note that RNS may show incremental response on high-frequency stimulation in some presynaptic (VAMP1, UNC13A, SYT2, RPH3A) and one synaptic (LAMA5) CMS subtype [8,9]. The inheritance patten in CMS is autosomal recessive except for dominant pattern seen in slow channel syndromes, SNAP25, PURA and some SYT2 patients [1^▪▪^]. Here, we highlight the widening phenotype spectrum associated with gene mutations that can not only have an effect of the NMJ but also show systemic and brain involvement (Table 2), and on current and emerging therapeutic strategies for CMS. 

**Box 1:**
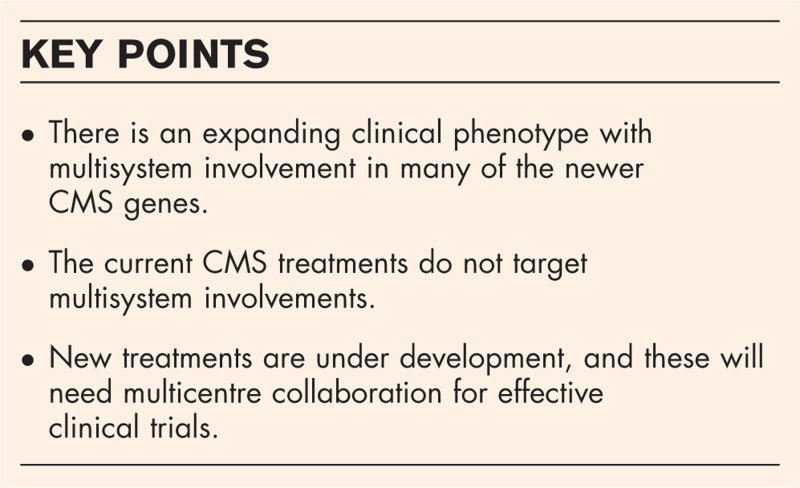
no caption available

**Table 1:** Summary of congenital myasthenia syndrome genes

Presynaptic	
Defects in acetylcholine synthesis and recycling	*CHAT, SLC5A7, SLC18A3, PREPL*
Vesicular exocytosis	*SYT2, SNAP25B, VAMP1, MUNC13-1, RPH3A*
Axonal transport	*MYO9*
Synaptic
Endplate AChR deficiency	*COLQ*
Defects in collagen	*COL13A1*
Defect in laminin	*LAMA5, LAMB2*
Postsynaptic	
Primary AChR deficiency	*CHRNA, CHRNB, CHRND, CHRNE*
AChR kinetics defects (with or without deficiency): Fast channel syndrome and Slow channel syndrome	*CHRNA, CHRNB, CHRND, CHRNE* *CHRNA, CHRNB, CHRND, CHRNE*
Escobar	*CHRNG*
Sodium channel dysfunction	*SCN4A*
AChR-clustering defects Agrin RAPSN DOK7 LRP4 MUSK CHRND	*AGRN* *RAPSN* *DOK7* *LRP4* *MUSK* *CHRND*
Defects in glycosylation	*ALG2, ALG14, DPAGT1, GFPT1, GMPPB*
Defects in cytoskeleton	*PLEC*
Defects in mitochondrial function	*SLC25A1*
Defects in translational regulation	*PURA*
Defects in nuclear structure	*TOR1AIP1*
Transcription regulation	*CHD8*
Defects in nAChR anchoring to cytoskeleton	*MACF1*

AChR, acetylcholine receptor.

**Table 2:** Nonneuromuscular junction involvement in congenital myasthenia syndrome genes reported in the literature

Gene	Brain	Facial	Skeletal	Skin	Renal	Other neuromuscular	Gastrointestinal	Other
*CHAT*	Global developmental delay MRI brain- delayed myelination, thin corpus callosum	–	–	–	–			
*SLC5A7*	Global developmental delay Learning difficulties Seizures Abnormal EEG MRI Brain – atrophy	Dysmorphic features	–	–	–			
*SLC18A3*	Global developmental delay MRI brain- atrophy						Necrotising enterocolitis	
*SNAP 25*	Global developmental delay Ataxia Epilepsy	Dysmorphic features						
*SLC25A1*	Learning difficulties Epilepsy MRI- agenesis of corpus collosum							Optic nerve hypoplasia D-2-hydroxyglutaric and L-2- hydroxyglutaric aciduria
*UNC13A*	MRI – thin corpus callosum	Dysmorphic features						
*LAMB2*		Microcoria						Congenital nephrotic syndrome
*LAMA5*	MRI brain- atrophy Facial tics	Dysmorphic features, myopia					Inflammatory bowel disease	
*SYT2*						Neuropathy		
*MYO9A*	Global developmental delay							
*PREPL*	Global developmental delay				Cystinuria			
*COL13A1*	Mild-to-severe learning difficulties	elongated face, low-set ears, micrognathia and high-arched palate	Pectus carinatum, Moderate to severe Scoliosis with kyphosis, mild spinal rigidity					
*RAPSN*		Facial dysmorphism	Arthrogryposis					
*Glycosylation genes*	Learning difficulties					Raised creatine kinase Muscle biopsy – dystrophic changes, tubular aggregates		
*PLEC*				Epidermolysis bullosa		Muscular dystrophy		
*PURA*	Global developmental delay Epilepsy							
*LRP4*			Syndactyly and brachydactyly in upper and lower limbs					Congenital diaphragmatic hernia, Pulmonary hypertension

All features are not reported in all patients.

Historically, CMS has been broadly classified, based on the location of the affected protein or the site of functional impairments, into presynaptic, synaptic and postsynaptic CMS subtypes. Identification of mutations in ubiquitously expressed proteins that potentially affect functions at all these locations, or proteins that are derived from the presynaptic side of the NMJ but have their effect on the postsynaptic side can confuse these categories, but nevertheless they remain useful for understanding characteristics of the different CMS subtypes.

## PRESYNAPTIC CONGENITAL MYASTHENIA SYNDROMES

Presynaptic CMS were reported to be the least common CMS subtype but there is now a growing number of gene mutations in proteins associated with release of acetylcholine (Ach) from the motor nerve terminal [10^▪^]. The clinical phenotype is usually early onset at birth/neonatal or infancy with varying severity of respiratory involvement (recurrent apnoea and or respiratory dependence), bulbar weakness, hypotonia, with or without muscle weakness. They can be divided into two broad categories, those involved in the synthesis and recycling of ACh (choline acetyltransferase CHAT), vesicular acetylcholine transporter SLC18A3, the high-affinity choline uptake transporter SLC5A7, and the mitochondrial citrate transporter, SLC25A1; and those involved in vesicular exocytosis, largely made up of soluble N-ethylmaleimide-sensitive factor attachment (SNARE) protein complex [synaptosomal-associated protein 25, SNAP25B, synaptobrevin 1 (also known as vesicle-associated membrane protein 1, VAMP1)], MUNC13-1 (*UNC13A*) and synaptotagmin 2 (SYT2). SYT2 acts as a calcium sensor to trigger vesicular release and dominant mutations in SYT2 can cause Lambert–Eaton-like features at the NMJ but are mainly seen to have a mild distal neuropathy phenotype, whereas biallelic mutations in SYT2 usually result in early-onset severe phenotype. As vesicular exocytosis is a CMS common process within many organs, and especially within the central nervous system, it is not surprising that gene defects that affect presynaptic neurotransmitter release at the NMJ can cause a wider phenotypic spectrum. Other proteins where mutations are thought to have presynaptic effects include PREPL, which encodes for the protein prolyl-oligopeptidase, and is proposed to be involved in trafficking of the vesicular ACh transporter at the NMJ and MYO9A, encoding myosin-IXA, that is involved in neuronal branching and axonal transport. RPH3A encodes for Rabphilin 3a, recently reported in a single case and is considered to be involved in synaptic vesicle regulation [9]. Phenotypic features distinct from myasthenia for mutations in many of these genes are highlighted in Table 2.

## Synaptic CONGENITAL MYASTHENIA SYNDROMES

The arrangement of proteins located in the synaptic cleft of the NMJ is poorly understood but mutations have been identified in a number that can have functional and/or structural roles. COLQ encodes for collagenic tail and binding domains, which anchors the asymmetric form of acetylcholinesterase to the basal lamina. Views on the exact binding partners for COLQ vary, but the most recent target put forward is LRP4 [11], which would tie in with a role for COLQ in influencing postsynaptic structures as well as ensuring (through acetylcholinesterase) the efficient breakdown of ACh. Reduced COLQ results in the persistence of ACh within the synaptic cleft, causing desensitization of endplate AChR with consequently reduced end-plate potentials, restricting the activation of the postsynaptic sodium channels (SCN4A). COLQ CMS also leads to prolonged and excessive AChR signalling, leading to AChR declustering, NMJ destabilisation, and myopathic changes. Clinical phenotype in COLQ CMS can vary from severe neonatal onset with hypotonia, muscle weakness, respiratory and bulbar involvement to milder phenotypes with late childhood or adult-onset proximal weakness [12]. Other CMS-associated synaptic gene mutations are mainly thought to have their effects on synaptic structure. LAMB2 encodes laminin beta 2, and LAMA5 encodes for laminin alpha 5, both of which are key components of the basal lamina and have a crucial role in organization of synaptic vesicle release sites known as active zones, promoting synaptic interactions, development and maturation of the postsynaptic region. In both LAMB2 and LAMA5 CMS, there are singleton cases reported, presenting with neonatal onset hypotonia, muscle weakness, bulbar, ocular and respiratory involvement with congenital nephrotic syndrome in LAMB2 patient and facial tics in LAMA5 patient [13,14]. LAMB2 mutations are recognized to cause Pierson syndrome, which presents with congenital nephrotic syndrome, ocular abnormalities like microcoria, developmental delay, hypotonia and muscle weakness and LAMA2 mutations cause focal segmental glomerulosclerosis, hearing loss, craniofacial dysmorphism and limb development defects [15]. COL13A1 (10q22.1) encodes the alpha-1 chain of the nonfibrillar transmembrane collagen type XIII, an essential component for development and maturation of both presynaptic and postsynaptic regions. The clinical phenotype is early onset with hypotonia, respiratory difficulties, bulbar difficulties, ptosis (nonfatigable) facial dysmorphology with skeletal abnormalities, predominantly axial weakness and cognitive difficulties [16].

## POSTSYNAPTIC CONGENITAL MYASTHENIA SYNDROMES

Postsynaptic CMS account for the majority of CMS cases. The predominant site of mutations is within the muscle acetylcholine receptor (AChR), but these may have varying underlying pathogenic molecular mechanisms. These mainly result in a deficiency of AChR at the motor endplate or abnormal kinetics of the AChR ion channel or a combination of the two. Within the AChR-encoding genes (*CHRNA1*, *CHRNB1*, *CHRND*, *CHRNE* and *CHRNG*) deficiency mutations in CHRNE are most common as this encodes the subunit for the adult muscle form of the AChR and null mutations in this gene can be partially compensated by the maintained expression of the fetal isoform γ subunit encoded by *CHRNG*. Over time, more deficiency mutations within the other subunits (*CHRNA1*, *CHRNB1*, *CHRND*) are coming to light and demonstrate varying levels of severity [17]. Kinetic mutations can be present in all four of the different subunits that make up the adult AChR and again show varying degrees of severity. The clinical phenotype of AChR deficiency because of CHRNE mutations occurring is of early-onset bilateral ptosis, ophthalmoparesis and generalized limb weakness. Bulbar dysfunction and recurrent respiratory illnesses can be seen in young children in the first decade but these tend to resolve, and overall CMS features remain stable in adulthood. The defects in the AChR channel-opening kinetics can lead to fast channel CMS where there is reduced AChR channel open time and consequently cause a reduction in postsynaptic depolarization and failure to trigger muscle action potential or slow channel syndrome where there is prolonged AChR opening causing a secondary endplate myopathy. Most fast channel CMS found in the UK tend to be severe with onset at birth, generalized weakness, ptosis, ophthalmoplegia, bulbar dysfunction and life-threatening respiratory crises [18]. The phenotype in slow channel CMS is more varied, though in early years, they frequently respond well to treatment. Onset can be from birth, childhood to adulthood and weakness is generalized or may selectively involve of scapular, cervical and distal upper limb muscles with or without ocular involvement [19]. AChR deficiency also results from mutations in RAPSN, the protein that anchors the tight aggregation of AChR molecules in the postsynaptic membrane, although the resulting phenotype differs from mutations within the AChR itself or from within the AGRN-signalling pathway. RAPSN mutations present at birth or in infancy with hypotonia, weakness, ptosis, bulbar difficulties and recurrent respiratory crisis, which can be life-threatening. These crises tend to be frequent in infancy and early childhood and then improve, overall patients tend to have minimal or mild weakness in adult life.

Agrin-induced AChR clustering pathway related CMS: the Agrin (AGRN)-LRP4-MUSK-DOK7 signalling pathway is crucial for the formation and maintenance of the NMJ. Agrin, which is secreted from the nerve terminal into the synaptic cleft binds to the LRP4 (low-density lipoprotein receptor-related protein 4) on the postsynaptic membrane and activates MUSK (muscle skeletal receptor tyrosine kinase). Activated MUSK signalling is amplified by the interaction with DOK7 (downstream of tyrosine kinase 7) and leads to the clustering and stabilization of AChR in the postsynaptic membrane. Mutations in *AGRN*, *LRP4*, *MUSK* and *DOK7* present with limb-girdle pattern of weakness with varying severity, often with some ptosis but minimal eye movement abnormality, though there is marked bulbar/respiratory involvement in some cases.

## CONGENITAL MYASTHENIA SYNDROMES DUE TO GLYCOSYLATION DEFECTS

Glycosylation is a ubiquitous process that is involved in protein folding, solubility, stability, assembly and intracellular transport and involves O-glycosylation and N-glycosylation. Glycosylation defects usually cause multiorgan disorders and include congenital disorders of glycosylation, congenital and limb girdle muscular dystrophy, and congenital myasthenic syndromes. Abnormal glycosylation results in reduced AChRs at the muscle endplates, leading to impaired neuromuscular transmission but it is still unclear as to why defects in this ubiquitous process cause a primary NMJ dysfunction. There are currently five genes known to cause CMS because of glycosylation defects all of whom are involved in the N-glycosylation pathway, they are *GFPT1*, *DPAGT1*, *ALG2*, *ALG14* and *GMPPB*. It is of note that these genes code for proteins at the early stages or feed in to the early stages of the N-linked pathway and so presumably the NMJ is highly sensitive to disruption at this stage. Clinical phenotype is of onset in childhood to adulthood with limb-girdle weakness without usually significant eye, respiratory or bulbar involvement. Other features include raised creatine kinase, muscle biopsy in some cases showing dystrophic changes (*GMPPB)* and tubular aggregates (*GFPT1*, *DPAGT1* and *ALG14*). Mild-to-moderate cognitive difficulties have been reported in some cases.

## OTHER CONGENITAL MYASTHENIA SYNDROME SUBTYPES

Several other genes are reported to associate with a CMS phenotype. *TOR1AIP1* (*torsinA-interacting protein 1* gene) encodes the inner nuclear membrane protein lamin-associated protein 1 (LAP1). Whereas nuclear membrane proteins are associated with a number of muscle disorders, this is the first where mutations impair NMJ function, although the pathogenic molecular mechanism has yet to be determined. The CMS phenotype described is of childhood-onset fatigable limb-girdle pattern muscle weakness without facial or bulbar involvement [20]. *PLEC1*, which encodes for plectin is a ubiquitously expressed intermediate filament-linking protein concentrated at sites of mechanical stress. *PLEC1* mutations result epidermolysis bullosa in isolation or in association with limb-girdle muscular dystrophy. *SCN4A* encodes the skeletal muscle voltage-gated sodium channel Nav1.4 and is a well recognized cause of channelopathies, including hyperkalemic and hypokalemic periodic paralysis, paramyotonia congenita and sodium channel myotonia with rare cases of SCN4A-related CMS reported. *PURA* gene encodes Pur-alpha, which is a ubiquitous protein that has RNA and DNA binding properties and is known to be associated with neurodevelopmental problems, a CMS phenotype has now been reported in some cases [21,22].

## TREATMENT

Currently there are no licensed drugs specifically for CMS treatment and all the drugs used are off-label, the doses and formulations used in CMS can be different to the licensed uses. Given that CMS is a heterogeneous disorder with many different underlying molecular mechanisms, the treatment response depends upon the subtype of CMS and treatment needs to be tailored for disease severity and patient response and can involve utilizing different combinations of the drugs. It is important to note that drugs that benefit some CMS types can worsen other subtypes (COLQ, DOK7 and Slow channel CMS worsen with acetylcholine esterase inhibitors) and hence in those awaiting genetic confirmation, empirical treatment must be with caution. The common drugs used are acetylcholinesterase inhibitors (Pyridostigmine), 3,4 diaminopyridine (3,4-DAP), Salbutamol and Ephedrine. Fluoxetine and Quinidine are used in slow channel CMS.

Acetylcholinesterase inhibitors slow the breakdown of acetylcholine in the synaptic cleft and hence prolong its activity. Pyridostigmine (Mestinon) is most commonly prescribed and is the first-line treatment in presynaptic CMS, AChR deficiency CMS, fast channel CMS and CMS due to glycosylation defects. The main side effects include nausea, abdominal cramps, diarrhoea, increased sweating and respiratory secretions. Side effects are well controlled by antimuscarinic agents like propantheline. Intramuscular neostigmine may be used during apnoeic episodes in infants and children.

3,4-DAP is a potassium channel blocker and works on the presynaptic nerve terminal by prolonging the motor nerve action potential, and hence increasing the release of acetylcholine into the synaptic cleft. Common side effect is perioral and peripheral paraesthesia. Seizures have been reported as a rare side effect of 3,4-DAP when used in the treatment of Lambert–Eaton myasthenic syndrome though rarely seen in clinical practice. It is used as add on treatment in AChR deficiency, Fast channel CMS and RAPSN among the common CMS subtypes.

Fluoxetine and Quinidine act as blockers of the AChR ion channel when in the open state and are used only in slow-channel CMS. Quinidine can cause cardiac conduction defects and both Quinidine and Fluoxetine can prolong QT interval at high doses. Fluoxetine side effects reportedly include suicidal ideation so patients should be monitored.

Beta 2-adrenergic receptor agonists include Ephedrine, and Salbutamol (Albuterol in the USA) are treatment of choice in DOK 7 and COLQ CMS. They are proposed to stabilize the synaptic structure and hence the effects can take several months to have an optimal effect. They also can have role as add-on treatment to pyridostigmine in AChR deficiency CMS and RAPSN CMS. Baseline ECG, BP and potassium level is recommended prior to initiation, after each dose increase and during intercurrent illness. Side effects can include exacerbating muscle cramps (especially in DOK7), hypotension, tachycardia and hypokalaemia particularly at high doses. High doses should be prescribed with caution because of possible adverse effects, such as hypertension, anxiety, insomnia and palpitations.

### Recent developments in therapeutics

**FIGURE 2:**
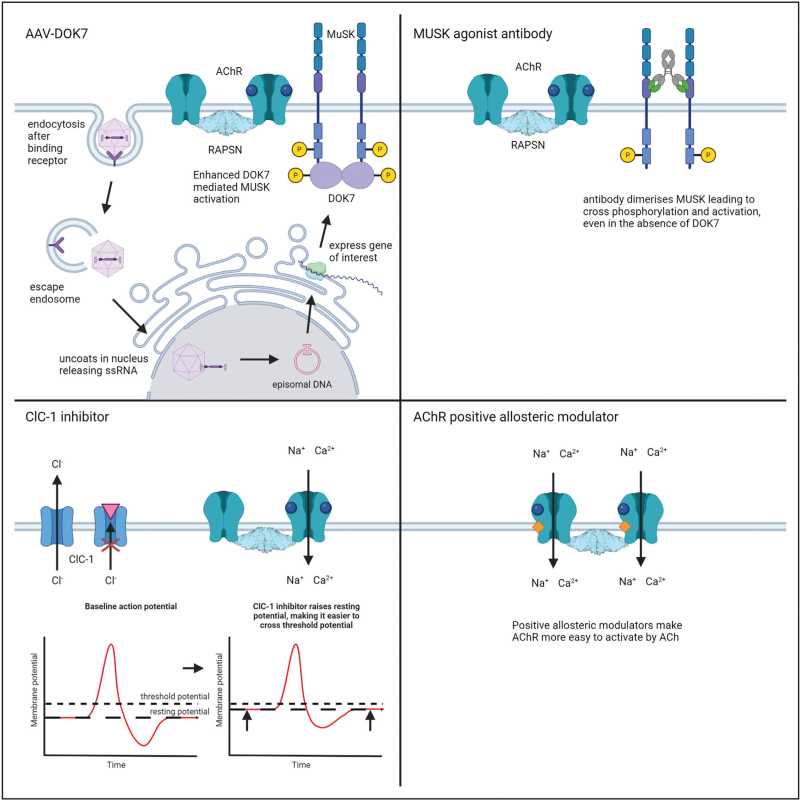
Therapeutic mechanisms of potential new treatments of congenital myasthenia syndromes – adenoassociated viral DOK7 replacement therapy, humanized MUSK agonist antibody therapy, ClC-1 chloride channel inhibitor treatment, muscle nicotinic acetylcholine receptor-positive allosteric modulator treatment.

Several studies have published promising data from preclinical trials on novel therapeutics for CMS (Fig. 2). These include using adeno-associated viruses (AAV) to deliver gene replacement therapies for treating deficiencies in *DOK7*, *COLQ* and *CHAT*, as well as a MUSK agonist antibody therapy that rescued a DOK7-CMS mouse model. Both the *AAV9*–*DOK7* gene therapy and the MUSK agonist antibody effectively rescued mouse models that harbour the most common mutation found in patients DOK7 c.1124_1127dupTGCC, making the mice as strong as wildtype littermates [23,24]. Similarly, an *AAV8–COLQ* gene therapy restored ColQ-deficient mice to [25]; and an *AAV9-CHAT* treatment rescued a mouse model *CHAT* deficiency to normal strength and neuromuscular signalling [26^▪▪^]. New therapeutic strategies aimed at improving neuromuscular transmission in myasthenia gravis by either inhibiting chloride channels to lower the safety factor [27^▪▪^], or by positive allosteric modulation of nAChR activity [28^▪^] are intriguing as these strategies may also be applicable for treating a variety of subtypes of CMS. Natural history studies are now crucial to determine the most appropriate outcome measures and design for clinical trials.

### Looking to the future

Improved DNA-sequencing technology has made it cheaper and easier to conduct whole-exome and whole-genome sequencing, which quickly expanded the number of genes associated with CMS. However, it remains challenging to experimentally determine whether genetic variants of unknown significance (VUS) are pathogenic, particularly missense variants and variants in noncoding regions of genes. Recent developments in bioinformatics, particularly using artificial intelligence and machine learning, have produced a number of tools that look to evaluate variants based on their position within the gene structure (such as CADD and DANN), and how well conserved the change is in evolution (EVE) and their effect on splicing (SpliceAI). Núñez-Carpintero *et al.*[29^▪^] recently published a multilayered workflow combining genetics and metabolomics data to elucidate the molecular determinates of disease severity. A notable alternative approach recently published by Deepmind is Alphamissense, which uses their Alphafold2 structural prediction software and boasts a 90% accuracy in predicting the pathogenicity of missense variants deposited in ClinVar [30^▪^]. Others have combined sequenced-based approaches with the 3D position information to predict protein stability [31]. Several recent publications of near atomic resolution structures of electric ray muscle type nAChR in resting and desensitized conformations have improved our understanding of the structure–function relationship of these channels, helping us understand how mutations in the channel change its function [32–34].

## CONCLUSION

The spectrum of CMS disorders is still expanding with multisystem involvement in many of the newly identified CMS-associated genes, particularly presynaptic gene defects. This has important implications on treatment strategies as the current CMS treatments are unlikely to affect the systemic symptoms. The newer treatments in the pipeline are likely to be in clinical trials before long and there is a need to ensure that there are robust reliable outcome measures. Finally, as many CMS are treatable, expanding newborn screening programmes globally would be ideal for the early diagnosis of CMS and reducing the mortality and long-term morbidity that can results from diagnostic delays.

## Acknowledgements

*We would like to thank all the CMS patients who have greatly facilitated our studies over many years.*

### Financial support and sponsorship

*None.*

### Conflicts of interest

*S.R. served on advisory board for Novartis, Sarepta, Argenx and Roche; is an investigator in clinical trials for Sarepta, Roche, Wave, Genetx, Argenx, Inois and Santhera associated with CMS; has received speaker fees for educational meetings from Novartis and Roche. Only discussions with Argenx are associated with congenital myasthenic syndrome. Y.Y.D. holds MRC fellowship MR/S007180/1; was the holder of ArgenX grant number: ARGX-NC-173-SP; and undertook a project for AMPLO Biotechnology.*

## REFERENCES AND RECOMMENDED READING

Papers of particular interest, published within the annual period of review, have been highlighted as:

▪ of special interest
▪▪ of outstanding interest
